# Supplementary material for: Correction: Incidence of Hepatocellular Carcinoma in Texas Latinos, 1995–2010: An Update
Source: PLoS One. 2014 Jul 21;9(7):e103693. doi: 10.1371/journal.pone.0103693 (PMC4105531; doi:10.1371/journal.pone.0103693)
Supplement: File S1 — Originally published, uncorrected article. (PDF) [file pone.0103693.s001.pdf]

OPEN ACCESS Freely available online

PLOS | ONE

Incidence of Hepatocellular Carcinoma in Texas Latinos, 1995–2010: An Update

Amelie G. Ramirez[1], Edgar Munoz[2], Alan E. C. Holden[1], Rebecca T. Adeigbe[2], Lucina Suarez[2]

1 Institute for Health Promotion Research and Cancer Therapy and Research Center, University of Texas Health Science Center at San Antonio, San Antonio, Texas, United States of America, **2** Institute for Health Promotion Research, University of Texas Health Science Center at San Antonio, San Antonio, Texas, United States of America

Abstract

Background: A previous study showed Hepatocellular Carcinoma (HCC) rates to be higher among Latinos in Texas and highest among South Texas Latinos compared to other non-Hispanic whites (NHW) and other Latinos in the United States (U.S.). We used more recent data to assess trends in HCC among Texas Latinos and to reassess the elevated HCC incidence rate in Texas Latinos.

Methods: We used data from the U.S. SEER Program and the Texas Cancer Registry to calculate annual and 3-year moving average age-specific and age-adjusted HCC incidence rates, annual percent changes (APCs), and their corresponding 95% confidence intervals for Latinos and NHW in the U.S., Texas and South Texas.

Results: Texas Latino male and female incidence rates were 3.1 and 4.0 times higher than their NHW counterparts in SEER regions. Latino males and females in South Texas had the highest rates of HCC incidence overall; rate ratios were 3.6 and 4.2 among South Texas Latino males and females compared to SEER NHW counterparts. There are statistically significant increases in HCC incidence rates in all groups (Texas and South Texas Latinos and NHW groups) and across all age groups. The elevated HCC rates in Texas Latinos are consistent over the 1995–2010 period.

Conclusions: The incidence of HCC among Latinos in South Texas remains higher than elsewhere in the U.S. and warrants closer investigation of potential risk factors related to prevailing conditions unique to the population including higher obesity and diabetes rates, environmental, cultural and socioeconomic factors and possibly genetic predisposition.

Citation: Ramirez AG, Munoz E, Holden AEC, Adeigbe RT, Suarez L (2014) Incidence of Hepatocellular Carcinoma in Texas Latinos, 1995–2010: An Update. PLoS ONE 9(6): e99365. doi:10.1371/journal.pone.0099365

Editor: Anand S. Mehta, Drexel University College of Medicine, United States of America

Received January 17, 2014; **Accepted** May 13, 2014; **Published** June 10, 2014

Copyright: © 2014 Ramirez et al. This is an open-access article distributed under the terms of the Creative Commons Attribution License, which permits unrestricted use, distribution, and reproduction in any medium, provided the original author and source are credited.

Funding: The authors gratefully acknowledge the Cancer Center Support Group of the Cancer Therapy and Research Center at the University of Texas Health Science at Center San Antonio, an NCI-designated Cancer Center (grant # P30CA054174) and Redes En AcciÃ3n: The National Latino Cancer Research Network (U01 CA114657-05) for support of this study. The funders had no role in study design, data collection and analysis, decision to publish, or preparation of the manuscript.

Competing Interests: The authors have declared that no competing interests exist.

* E-mail: ramirezag@uthscsa.edu

Introduction

Hepatocellular Carcinoma (HCC) incidence rates continue to increase globally and in the United States (U.S.); incidence rates have historically been higher in Asia and West and Central Africa however surveillance trends indicate that HCC age-adjusted incidence rates have almost tripled in the U.S. over the past 20 years [1,2]. Others have postulated that the increase in HCC incidence is related to the changing risks associated with the Hepatitis B and C virus, cirrhosis, heavy alcohol consumption, diabetes, obesity and other metabolic syndromes [2,3]. The contraction and development of these HCC risks tend to be more prominent among minority racial and ethnic groups and immigrant groups [4–6].

In the U.S. HCC is the primary type of liver cancer diagnosed accounting for approximately 90% of all liver cancer cases [1]. Historical data from SEER and other registries show a long-standing discrepancy in the incidence of HCC between Latinos and NHW, with Latinos experiencing HCC rates that are twice as high as NHW [7]. SEER data however exclude a sizable proportion of the U.S. Latino population because Texas, which accounts for over one-fifth of the U.S. Latino population, is not included in the SEER registries. We have previously reported on the much higher HCC incidence rates of Latinos living in Texas and South Texas. For the period 1995–2006, HCC age adjusted incidence rates in South Texas Latinos were 10.6/100,000 compared to 9.5 and 7.5 for Latino in the rest of Texas and the U.S. and 3.1 and 2.9 among NHW in Texas and the U.S. [8].

We examined the trends in HCC incidence for Latinos in South Texas and Texas and reassessed the elevated relative risks among Texas Latinos using more recent data through 2010.

Methods

We obtained data from the U.S. SEER Program and the Texas Cancer Registry (TCR) [9] under Limited-Use Data Agreements between the authors and the sources. The Texas Cancer Registry, a statewide population-based registry, is Gold Certified by the North American Association of Central Cancer Registries as meeting the highest level data quality standards including case

completeness and timeliness of case reporting [10]. Informed consent was not required for de-identified data and thus this study was exempted from review by the University of Texas Health Science Center at San Antonio Institutional Review Board.

HCC incident cases from 1995 through 2010 were selected for Latino and NHW male and female residents of the 13 SEER registries (Connecticut, Hawaii, Iowa, New Mexico, Utah, and metropolitan Atlanta, Detroit, Los Angeles, San Francisco-Oakland, San Jose-Monterey, Seattle-Puget Sound, Rural Georgia and Alaska) ($n = 5{,}560$ Latino; 13,584 NHW); the state of Texas ($n = 6{,}656$ Latino; 7,597 NHW) and the 38 counties comprising South Texas ($n = 3{,}317$ Latino; 1,038 NHW). Cases were defined using SEER criteria (International Classification of Diseases (ICD-O-3 topography C22.0 and morphologies 8170–8175)) [11]; rates were determined using SEER population denominators adjusted for Hurricane Katrina but not for delay in case reporting [12]. Sixty percent of the SEER HCC and 65% of the TCR HCC cases were confirmed histologically. Ethnicity was defined using the North American Association of Central Cancer Registries (NAACCR) Hispanic/Latino Identification Algorithm, version 2 [13].

Using SEER*Stat software v 8.1.2 (SEER*Stat, National Institutes of Health) [12], we generated 1995–2010 average annual age-specific, age-adjusted, and three-year moving average HCC incidence rates, rate ratios (RR), annual percent changes (APCs) and 95% confidence intervals (CI) for Latino and NHW populations in the SEER, Texas and South Texas regions. APCs were derived using weighted least squares point-estimation; trends were tested for statistical significance using SEER*Stat.

Results

Table 1 shows the HCC incidence RR among Latinos in the U.S., Texas, and South Texas compared to NHW in SEER regions. U.S. SEER Latinos had 2.5 and 2.9 times the HCC incidence rates than NHW males and females, respectively. RR for Texas Latino male and female incidence rates were 3.1 (95% CI = 3.0, 3.2) and 4.0 (95%CI = 3.8, 4.2), respectively, compared to their NHW counterparts in SEER regions. Latino males and females in South Texas had the highest rates of HCC incidence overall; RR were 3.6 (95% CI = 3.5, 3.8) and 4.2 (95% CI = 3.9, 4.5) among South Texas Latino males and females, respectively. Notably, RR were also slightly increased among NHW living in Texas and South Texas, ranging from 1.1 to 1.3 (Table 1).

Figure 1 shows that the age-adjusted incidence rates of HCC among South Texas and Texas Latinos have been consistently higher than NHW throughout the 1995–2010 period. All groups, Latino and NHW show increasing rates of HCC over the period. These trends, based on the APC, are statistically significant among all groups (Table 2). Examining the APCs by age shows that these steady increases over time are occurring in all age groups, Latino and NHW. Across the ethnic groups, those in the age group 50–59 years experienced the highest increases.

Figure 2 shows the trends in Latino incidence rates for each age group as compared to the NHW in the SEER regions. The Latino gap in HCC incidence is consistent over the years for each age group; the South Texas rates remain highest in each age group and year without discernible improvement.

Discussion

We reassessed current trends in HCC incidence and the recent data show that the elevated rates in Latinos have not improved over time. South Texas Latinos have the highest HCC rates in the country with rates 3 to 4 times higher than NHW in SEER

Table 1. Incidence Rates[1] and Rate Ratios (RR) of HCC in Latinos from US SEER, Texas and South Texas, 1995-2010.

	US SEER			Texas			South Texas		
	N	Rate[1] (95% CI)	RR[2] (95% CI)	N	Rate (95% CI)	RR (95% CI)	N	Rate (95% CI)	RR (95% CI)
Hispanic									
Male	4,143	13.6 (13.2–14.1)	**2.48 (2.39–2.57)**	4,849	17.2 (16.7–17.7)	**3.13 (3.02–3.24)**	2,423	19.9 (19–20.7)	**3.61 (3.45–3.77)**
Female	1,417	4.2 (4–4.4)	**2.92 (2.74–3.11)**	1,807	5.8 (5.5–6.1)	**4.01 (3.78–4.24)**	894	6.0 (5.6–6.4)	**4.16 (3.86–4.48)**
Total	5,560	8.4 (8.2–8.7)	**2.55 (2.47–2.63)**	6,656	10.9 (10.6–11.2)	**3.29 (3.19–3.39)**	3,317	12.1 (11.7–12.5)	**3.64 (3.5–3.78)**
NHW									
Male	10,343	5.5 (5.4–5.6)	**1.00 -**	5,777	6.1 (6–6.3)	**1.11 (1.08–1.15)**	795	7.1 (6.6–7.6)	**1.28 (1.19–1.38)**
Female	3,241	1.4 (1.4–1.5)	**1.00 -**	1,820	1.6 (1.6–1.7)	**1.14 (1.08–1.21)**	243	1.8 (1.6–2)	**1.23 (1.08–1.41)**
Total	13,584	3.3 (3.3–3.4)	**1.00 -**	7,597	3.7 (3.6–3.8)	**1.12 (1.09–1.15)**	1,038	4.3 (4–4.6)	**1.29 (1.21–1.37)**

[1]Rates per 100,000 and age-adjusted to the 2000 US Standard Population (19 age groups).
[2]Rate Ratios calculated using US SEER NHW (non-Hispanic whites) groups as reference.
doi:10.1371/journal.pone.0099365.t001

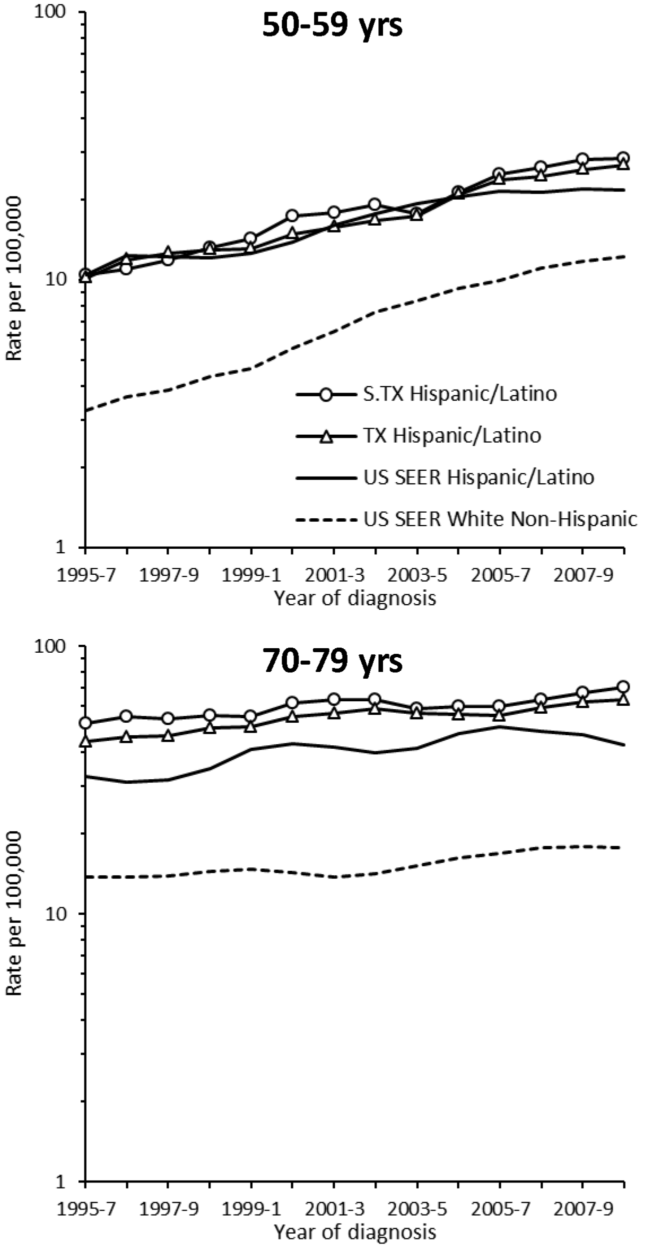

Figure 1. Annual Age-adjusted incidence rates of hepatocellular carcinoma by ethnicity, 1995–2010. Annual age-adjusted incidence of HCC increased over the study period and was highest among South Texas Latinos. HCC incidence for the three Latino populations was consistently higher than for non-Hispanic whites (NHW).
doi:10.1371/journal.pone.0099365.g001

regions. This high elevation in rates among South Texas Latinos are consistent across all age groups and have remained constant over the last 15 years. To date, no studies have determined the cause of the high rates of HCC among South Texas Latinos, nor the reasons for the steady increase in HCC over the years.

Latinos in South Texas represent a unique population, suffering from more obesity, diabetes and HCC than other Latinos. One-third of South Texas adults are obese compared to 29% and 27% for the rest of Texas and the U.S. and 12% suffer from diabetes compared to 9% for the rest Texas and the U.S. [14]. Proximity to the Mexico border may also expose the population to other threats including environmental contamination and hazards. South Texas Latinos are mainly of Mexican descent [15] and about a third of the Mexican American gene pool is derived from Native American sources [16]. The differential rates in HCC between Latinos in Texas and those living elsewhere in the U.S. may be due to differences in population composition of these Latino subgroups. In South Texas, 94% of Latinos are of Mexican origin while the Mexican-origin population of counties comprising the SEER 13 registries is 70% [17].

Before practical interventions for HCC are implemented, the true causes of the rising and higher HCC rates in South Texas must be understood. Recent federal funding regarding HCC epidemiology provides some encouragement in this regard, but the overall decline in such funding is of concern. Understanding the causes of increasing HCC in South Texas is critical not only for

Table 2. Annual percent change (APC) of HCC incidence[1] from 1995 to 2010 by age for US SEER, Texas and South Texas.

	US SEER	Texas	South Texas
	APC (%) (95% CI)	APC (%) (95% CI)	APC (%) (95% CI)
Hispanic			
All ages	3.6* (2.7–4.5)	3.2* (2.6–3.9)	2.9* (2.3–3.5)
50–59	5.7* (4.2–7.2)	7.8* (6.3–9.3)	8.0* (6–10)
60–69	3.5* (2.2–4.9)	2.6* (1.1–4.2)	1.6 (−0.3–3.5)
70–79	2.4* (0.6–4.2)	2.5* (1.6–3.3)	2.2* (0.9–3.4)
80+	3.6* (1.1–6.3)	1.6 (–0.3–3.6)	2.0* (0.3–3.8)
WNH			
All ages	4.8* (4.3–5.3)	5.5* (4.8–6.2)	4.7* (3.3–6.1)
50–59	10.6* (9.2–12)	12.4* (10.9–13.9)	12.3* (9.1–15.6)
60–69	5.0* (3.7–6.3)	4.7* (3.1–6.3)	3.4* (1–5.9)
70–79	2.1* (1.4–2.9)	2.7* (1.4–4.1)	0.5 (–1.6–2.7)
80+	3.0* (1.7–4.2)	2.7* (1.5–4)	3.2 (−0.3–6.9)

[1]Incidence rates are age-adjusted for all ages and unadjusted for specific age groups.
*Significantly increasing trend ($p < .05$).
APC = Annual Percent Change.
CI = Confidence Interval.
doi:10.1371/journal.pone.0099365.t002

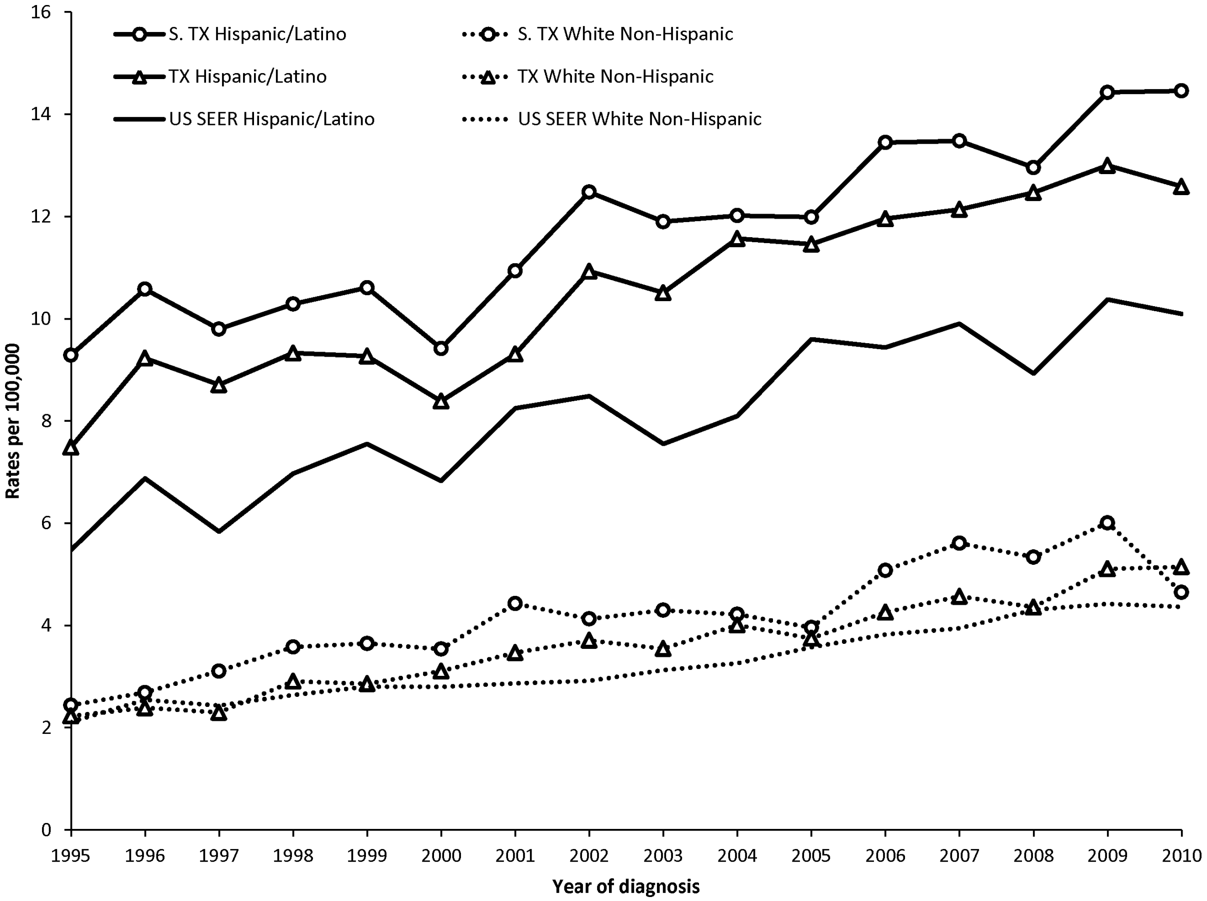

Figure 2. Age-specific incidence trends of hepatocellular carcinoma. Age-specific incidence of HCC was consistently higher among Latinos than among non-Hispanic whites (NHW). Each point was estimated as a 3-yr moving average.
doi:10.1371/journal.pone.0099365.g002

developing HCC interventions but also for identifying high risk individuals so that they may be screened and treated with the best available care.

Acknowledgments

The authors gratefully acknowledge the staff at the Texas Cancer Registry, Texas Department of State Health Services, for providing the cancer incidence data and technical support. Finally, we would like to express our gratitude to Mr. Kipling Gallion, Deputy Director of the Institute for Health Promotion Research for their insight and encouragement throughout this effort.

Author Contributions

Conceived and designed the experiments: AGR EM ACH LS. Performed the experiments: EM ACH LS. Analyzed the data: EM ACH LS. Contributed reagents/materials/analysis tools: AGR EM ACH LS. Wrote the paper: AGR EM ACH RTA LS.

References

1. Altekruse SF, McGlynn KA, Reichman ME (2009) Hepatocellular carcinoma incidence, mortality, and survival trends in the United States from 1975 to 2005. Journal of clinical oncology: official journal of the American Society of Clinical Oncology 27: 1485–1491.
2. Welzel TM, Graubard BI, Quraishi S, Zeuzem S, Davila JA, et al. (2013) Population-attributable fractions of risk factors for hepatocellular carcinoma in the United States. The American journal of gastroenterology 108: 1314–1321.
3. Saunders D, Seidel D, Allison M, Lyratzopoulos G (2010) Systematic review: the association between obesity and hepatocellular carcinoma - epidemiological evidence. Alimentary pharmacology & therapeutics 31: 1051–1063.
4. Artinyan A, Mailey B, Sanchez-Luege N, Khalili J, Sun C-L, et al. (2010) Race, ethnicity, and socioeconomic status influence the survival of patients with hepatocellular carcinoma in the United States. Cancer 116: 1367–1377.
5. Hu DJ, Xing J, Tohme RA, Liao Y, Pollack H, et al. (2013) Hepatitis B testing and access to care among racial and ethnic minorities in selected communities across the United States, 2009-2010. Hepatology (Baltimore, Md) 58: 856–862.
6. El-Serag HB (2004) Hepatocellular carcinoma: Recent trends in the United States. Gastroenterology 127: S27–S34.
7. Mendez-Sanchez N, Zamora-Valdes D, Vasquez-Fernandez F, Uribe M (2007) Hepatocellular carcinoma in Hispanics. Ann Hepatol 6: 279–280.
8. Ramirez AG, Weiss NS, Holden AEC, Suarez L, Cooper SP, et al. (2012) Incidence and risk factors for hepatocellular carcinoma in Texas Latinos: implications for prevention research. PloS one 7: e35573.
9. Surveillance Epidemiology and End Results (SEER) Program SEER*Stat Database: Incidence - SEER 13 Regs Research Data, Nov 2012 Sub (1992–2010) Katrina/Rita Population Adjustment - Linked To County Attributes - Total U.S., 1969–2011 Counties, National Cancer Institute, DCCPS, Surveillance Research Program, Surveillance Systems Branch, released April 2013, based on the November 2012 submission.
10. Texas Department of State Health Services Texas Cancer Registry (www.dshs.state.tx.us/tcr) SEER*Stat Database, 1995–2010 Limited-Use Incidence, Texas statewide, Texas Department of State Health Services, created June 2013, based on NPCR-CSS Submission, cut-off 11/30/12.
11. Fritz A, Percy C, Jack A, Shanmugaratnam K, Sobin L, et al. (2000) International Classification of Diseases for Oncology, 3rd ed (ICD-O-3). Geneva: World Health Organization. 240 p.
12. Surveillance Research Program National Cancer Institute SEER*Stat software (www.seer.cancer.gov/seerstat) version 8.1.2.
13. NAACCR: North American Association of Central Cancer Registries (2008) Guideline for Enhancing Latino/Latino Identification: Revised NAACCR Latino/Latino Identification Algorithm [NHIA v2]. North American Association of Central Cancer Registries.
14. Ramirez A, Thompson I, Vela L (2013) The South Texas health status review a health disparities roadmap. Cham; New York: Springer.
15. Institute for Health Promotion Research, UT Health Science Center at San Antonio (2007) South Texas Health Status Review.
16. Reed TE (1974) Ethnic classification of Mexican-Americans. Science 185: 283.
17. U.S. Census Bureau 2008–2012 American Community Survey.
